# The *Streptococcus mutans* collagen-binding protein Cnm enhances early biofilm formation with *Candida albicans*

**DOI:** 10.1128/aem.01046-26

**Published:** 2026-06-30

**Authors:** Callahan Katrak, Leslie Bautista, Lucy Pepe, Jeffery Fairman, Jacqueline Abranches

**Affiliations:** 1 Department of Oral Biology, University of Florida College of Dentistry 164889, Gainesville, Florida, USA; 2 Vaxcyte Inc. 681082 , San Carlos, California, USA; The University of Tennessee Knoxville, Knoxville, Tennessee, USA

**Keywords:** cross-kingdom interactions, biofilm ecology, *Candida albicans*, *Streptococcus mutans*, oral microbiology, collagen binding, adhesins, microbial interactions

## Abstract

*Streptococcus mutans* strains expressing the collagen-binding protein Cnm are strongly associated with severe and recurrent dental caries, yet the mechanistic basis for their clinical enrichment alongside *Candida albicans* remains unclear. Here, we investigated whether Cnm contributes to early cross-kingdom interactions that promote dual-species biofilm development. Using purified proteins, we found that *C. albicans* binds robustly to Cnm at levels comparable to those of glucosyltransferase B (GtfB) and higher than those of other *S. mutans* adhesins tested. Preincubation of collagen with Cnm inhibited fungal binding, indicating that Cnm cannot simultaneously engage collagen and *C. albicans*, and suggesting competition for the same or adjacent binding regions. Cnm expression significantly enhanced coaggregation with *C. albicans* in both collagen-free and collagen-rich environments. In early attachment assays, Cnm increased *S. mutans* adherence to collagen-coated surfaces and promoted *C. albicans* recruitment on uncoated surfaces, consistent with ligand-dependent binding specificity. Pre-coaggregation in saliva further enhanced the ability of Cnm^+^ *S. mutans* to attach to *C. albicans*. At 24 h, Cnm expression increased biomass and *S. mutans* CFUs exclusively on collagen-coated surfaces, regardless of sucrose availability. Together, these findings identify Cnm as a dual-binding adhesin that associates with either collagen or *C. albicans,* depending on environmental context, thereby accelerating coaggregation, early colonization, and biofilm maturation. This mechanism provides a biological explanation for the co-enrichment of Cbp^+^ *S. mutans* and *C. albicans* seen in dental caries and highlights Cnm as a key mediator of cross-kingdom synergy in early biofilm formation.

**IMPORTANCE:**

Dental caries is a multifactorial and polymicrobial disease in which the consumption of fermentable carbohydrates favors acidogenic and aciduric microorganisms at the expense of beneficial commensal bacteria, creating a dysbiotic environment. *Streptococcus mutans* and *Candida albicans* establish a synergistic relationship that exacerbates dysbiosis, thereby promoting caries development and progression. The current paradigm of this cross-kingdom synergism centers on increased extracellular polysaccharide production and enhanced biofilm biomass in the presence of sucrose. Here, we show that the collagen-binding protein Cnm, produced by approximately 20% of *S. mutans* isolates, promotes interspecies co-aggregation with *C. albicans*, facilitating interspecies attachment and early biofilm formation. Our findings expand the current paradigm by demonstrating that Cnm recruits *C. albicans* to the developing biofilm. This interaction may play a crucial role in the stability and virulence of early biofilm communities, particularly under low-sucrose conditions.

## INTRODUCTION

Dental caries is one of the most prevalent chronic diseases globally, affecting over 2 billion individuals across all age groups, including approximately 500 million children (1, 2). This disease arises from dysbiosis in the complex microbial communities that form biofilms on tooth surfaces, largely driven by frequent consumption of fermentable dietary sugars. A key contributor to this dysbiosis is the gram-positive bacterium *Streptococcus mutans*, known for its acidogenic and aciduric properties and its ability to form robust biofilms, particularly in the presence of the cariogenic sugar sucrose (table sugar) (3). These traits enable *S. mutans* and other acid-tolerant organisms to outcompete the less acid-tolerant commensal species (4). However, the microbial component of caries development is not exclusively driven by bacteria, as increasing evidence indicates that fungi, especially the dimorphic yeast *Candida albicans*, can exacerbate the cariogenic process through its synergistic relationship with *S. mutans* (5).

Adhesion to and accumulation on the tooth surfaces are central to *S. mutans* biofilm formation and hence its pathogenicity. In sucrose-rich conditions, *S. mutans* synthesizes extracellular polysaccharides (EPS) via glucosyltransferase (Gtf) enzymes, facilitating adhesion to both the tooth surface and recruitment of microbes to the growing biofilm (3, 6). This EPS matrix supports biofilm maturation and contributes to localized acidification, driving enamel demineralization (7). Of particular relevance to *S. mutans–C. albicans* interactions, expression of *S. mutans* Gtfs is enhanced in the presence of *C. albicans*. Specifically, GtfB has been shown to directly bind to *C. albicans* surface, recognizing β-glucans and mannans present in *C. albicans* cell wall, and enhancing coaggregation between the two species in the presence of sucrose (8–10). Thus, even prior to surface attachment, coaggregation increases biofilm biomass at early timepoints and enhances the biofilm’s structural integrity, stress tolerance, and cariogenic potential (11). While Gtfs are the most thoroughly studied mediators of *S. mutans–C. albicans* synergism, sucrose is not always abundant in the oral cavity. Therefore, studying the contribution of sucrose-independent factors to *S. mutans–C. albicans* interactions may reveal additional synergistic mechanisms. For example, the *S. mutans* adhesin SpaP, which is part of a sucrose-independent mechanism of biofilm formation, was shown to contribute to increased biofilm biomass and recruitment of *C. albicans* in dual-species biofilms, suggesting that there is likely a multitude of uncharacterized cross-kingdom interactions (12).

Collagen is an ecologically meaningful substrate in the oral cavity because it constitutes ~18% of dentin and becomes exposed during caries progression, tooth wear, fracture, and gingival recession. Collagen is also part of the mucosa and can be exposed due to mucosal injury. These events create collagen-rich niches that differ chemically and physically from enamel surfaces and can selectively favor microorganisms capable of robust collagen binding. Thus, evaluating adhesin function under collagen-rich conditions reflects clinically relevant environments. Although all *S. mutans* strains possess adhesins that bind collagen, approximately 20% harbor bona fide collagen-binding proteins (Cbps), namely Cnm or Cbm (13–15). However, the majority of Cbp-positive (Cbp^+^) strains express Cnm, whereas Cbm is found in a small subset of strains (14). Both Cnm and Cbm contain highly conserved N-terminal collagen-binding domains (CBDs), and Cbm appears to be a domain-duplicated variant of Cnm with similar collagen-binding activity (14, 16). These Cbps promote adherence to collagen- and laminin-rich tissues in a sucrose-independent manner (14). Cnm^+^ strains are enriched in collagen-rich niches in the oral cavity, including dentin caries and root-surface lesions. Moreover, Cnm^+^ *S. mutans* is strongly associated with extra-oral infections such as infective endocarditis, where exposed collagen and laminin provide a selective advantage (16–19). Cnm is more commonly found in *S. mutans* serotypes e, f, and k than in the most prevalent serotype c (20). Notably, however, not all *cnm*-encoding strains exhibit strong collagen binding or high Cnm surface expression, suggesting that gene truncations, regulatory differences, post-translational processing, or cell-wall architecture can modulate Cnm function (21). These patterns indicate that collagen binding provides a context-dependent advantage in disease-associated environments, while potential fitness trade-offs or regulatory constraints may limit the prevalence and functional expression of Cnm across the broader *S. mutans* population.

Several clinical studies support the link between co-infection with *S. mutans* and *C. albicans* and more severe forms of dental caries, particularly in early childhood caries (ECC) (22–28). Furthermore, high salivary *C. albicans* counts are associated with high *S. mutans* levels and caries prevalence, highlighting a synergistic relationship that worsens disease outcomes (28, 29). Interestingly, unlike mucosal candidiasis where hyphae predominate, clinical imaging of dental plaque and carious lesions shows mixed *C. albicans* morphologies, including abundant yeast forms, consistent with the fact that acidic microenvironments typical of active caries inhibit the yeast-to-hypha transition (11, 30–32). Our previous work has shown that co-infection with Cbp^+^ *S. mutans* and *C. albicans* is strongly associated with early childhood caries cases that do not respond to current clinical interventions (22). Interestingly, total Mutans streptococci and yeast counts from dental plaque at deep dentin lesions were higher (8- and 13-fold, respectively) in children infected with Cbp^+^ *S. mutans* compared to those infected with Cbp⁻ *S. mutans* strains (22). Taken altogether, we hypothesized that Cnm produced by *S. mutans* enhances biofilm formation and promotes interspecies attachment and co-aggregation with *C. albicans*. This interaction may play a crucial role in the stability and virulence of early biofilm communities, particularly under low-sucrose conditions. Here, we investigate the role of Cnm in mediating the synergistic relationship between *S. mutans* and *C. albicans* during biofilm development.

## MATERIALS AND METHODS

### Growth conditions

Strains (Table 1), including UA159 and OMZ175 of *S. mutans*, their mutant derivatives, and SC5314 (CaWT) of *C. albicans,* were revived from 50% glycerol freezer stocks and streaked on Brain Heart Infusion medium (BHI) (Difco) agar. The antibiotics erythromycin (10 µg/mL) and kanamycin (1 mg/mL) were used in agar plates for maintenance or isolation of antibiotic-resistant transformants when necessary. BHI broth was routinely used for the preparation of overnight cultures that were later diluted with additional BHI to an OD_600 nm_ = 0.5. Colony-forming units (CFU) were determined by serial diluting and plating samples on Mitis Salivarius Agar with Bacitracin (MSB) for *S. mutans* recovery and Sabouraud Dextrose Agar (SAB) for *C. albicans* unless otherwise noted. *C. albicans* and clinical yeast starter cultures and SAB plates were incubated at 37°C in an ambient atmosphere. Cultures (including starter cultures, agar plates, and biofilm development) were incubated at 37°C in 5% CO_2_.

**TABLE 1:** *Streptococcus mutans* and *Candida albicans* strains used in this study^*a*^

Strain	Relevant characteristics	Source
UA159	*S. mutans* lab wild type *cnm*^−^	ATCC 700610
UA159+cnm	UA159 carrying chromosomally integrated pBGE::cnm Erm	(33)
OMZ175	*S. mutans* lab wild type *cnm*^+^	(34)
OMZ175Δcnm	OMZ175*Δcnm kan* via pALH124	(33)
CaWT	*C. albicans* lab wild type	ATCC MYA2876 (SC5314)
UA159+gfp	UA159 carrying chromosomally integrated pPMZ-Pveg-sfgfp *kan erm*	This study
UA159+cnm+gfp	UA159+*cnm* carrying chromosomally integrated pBGE::cnm Erm and pPMZ-Pveg-sfgfp *kan erm*	This study
OMZ175+gfp	OMZ175 carrying chromosomally integrated pPMZ-Pveg-sfgfp *kan erm*	This study
OMZ175Δcnm+gfp	OMZ175Δcnm+pPMZ-Pveg-sfgfp *kan erm*	This study

^*a*^ Genetically modified *S. mutans* strains used in this study carry chromosomal integrations. The cnm knock-in strain (UA159+cnm) contains pBGE::cnm integrated into the chromosome at the *gtfA* locus, and the cnm deletion in OMZ175Δcnm was generated using the integrative vector pALH124. Fluorescent derivatives were constructed using pPMZ-Pveg-sfgfp, which integrates at the mtlA–phnA locus. The erm and kan markers correspond to erythromycin resistance and kanamycin resistance cassettes encoded within the integrated constructs.

### *S. mutans* protein expression

Proteins representing known or putative adhesins with reported or predicted roles in host-surface binding or interspecies interactions were chosen to be expressed in a cell-free system to test *C. albicans*' ability to bind to a variety of *S. mutans* surface antigens. Genes encoding the proteins Cnm (1–308 amino acids; truncated protein full length: 545), SpaP (39–512 amino acids; truncated protein full length: 1563), PstS, GbpC, GtfD, GtfB, and WapA (R310N; amino acid substitution to prevent cleavage during protein expression) (Table 2) were synthesized at ATUM (Menlo Park, CA) and inserted into a custom vector using Ndel and SalI restriction sites. Each construct contained an N-terminal His12-tag and a TEV protease cleavage site. Proteins were expressed using the XpressCF^+^ Cell-Free Protein Synthesis platform in 200 mL reactions using the DASBox mini bioreactor system (Eppendorf). CFPS reaction mixtures were incubated with their respective plasmid (3 µg/mL) at 25°C for 10 h with constant stirring of 500 RPM, pH 7.2, and 30% dissolved oxygen. After incubation, each reaction was harvested and spun down at 7,500 RPM for 35 min at 4°C. The resulting supernatants were adjusted to 50 mM Tris, 150 mM NaCl, 7.5 mM imidazole, pH 8.0, using stock solutions and then filtered using a 0.45 µm membrane before purification.

**TABLE 2:** *S. mutans* proteins used in this study

*S. mutans* protein	Acronym	Function	UA159 gene locus	OMZ175 gene locus	Reference
Dps-like peroxide resistance protein	Dpr	Ferritin-like peroxide resistance protein; protects against oxidative stress by sequestering iron	SMU_RS02595	SMU109_RS07400	(35)
Collagen-binding protein	Cnm	Mediates binding to collagen and laminin	Not present	SMU109_RS01975	(34)
Surface protein antigen I/II	SpaP	Adhesin mediating attachment to tooth surfaces through binding salivary agglutinin (gp340); also known as P1, Antigen I/II, PAc	SMU_RS02900	SMU109_RS02425	(36)
Phosphate-binding protein	PstS	High-affinity phosphate uptake system component	SMU_RS05240	SMU109_RS06740	(37)
Glucan-binding protein C	GbpC	Glucan-binding protein C; contributes to sucrose-dependent biofilm formation	SMU_RS06360	SMU109_RS0109675	(38)
Wall-associated protein A	WapA	Cell surface protein which mediates bacteria–bacteria binding and enhances biofilm stability; also known as Antigen A	SMU_RS04535	SMU109_RS05125	(15)
Glucosyl-transferase D	GtfD	Synthesizes water-soluble glucans from sucrose	SMU_RS04210	SMU109_RS05680	(39)
Glucosyl-transferase B	GtfB	Produces insoluble α-1,3-linked glucan; known binding partner with *C. albicans*	SMU_RS04620	SMU109_RS00060	(10, 39)

Proteins Cnm (1–308), SpaP (39–512), PstS, GbpC, GtfD, GtfB, and WapA (R310N) were all purified using nickel-affinity chromatography.

Filtrates of Cnm (1–308), SpaP (39–512), GbpC, GtfD, and WapA (R310N) were each loaded onto a 5 mL His-Trap Excel column (Cytiva) and equilibrated with Buffer A1 (50 mM Tris, 150 NaCl, 10 mM imidazole, pH 8). The column was thoroughly washed with 10 CV Buffer A1, then 15 CV of Buffer A2 (50 mM Tris, 150 NaCl, 10 mM imidazole, pH 8, with 0.05% Triton X-100), and then again with 10 CV of Buffer A1. Bound protein was eluted in 8 CV Buffer B (50 mM Tris, 150 NaCl, 300 mM imidazole, pH 8). Eluted fractions were pooled and incubated with His-tagged TEV protease at a ratio of 1:5 TEV:protein overnight at 4°C dialyzing against 1× PBS, pH 8.0. The dialyzed cleavage product was loaded back onto the 5 mL His-Trap Excel column and equilibrated with dialysis buffer, and untagged protein was collected in the flow-through with 10 CV 1× PBS, pH 8. Flow-through fractions were pooled, then concentrated using a 10 kDa MWCO Amicon centrifugal filter (Millipore Sigma) in 1× PBS, pH 8.

PstS was purified using the same purification method, with the addition of 2M urea in Buffers A1, A2, and B. After elution, the fractions were incubated with TEV protease and dialyzed against 1× PBS, pH 8, 2M urea overnight at 4°C. The dialyzed sample was loaded back onto the column, and the untagged protein was collected in the flow-through with 10 CV dialysis buffer. These fractions were pooled, concentrated, and buffer exchanged in 1× PBS, pH 8, using a 10 kDa MWCO Amicon centrifugal filter (Millipore Sigma).

For GtfB, 0.1% Brij35 was added to the CFPS reaction mixture and Buffers A1, A2, and B following the same purification method. After elution, the protein was incubated with TEV protease and dialyzed against 1× PBS, pH 8, 0.1% Brij35 overnight at 4°C. The sample was then loaded back onto the column, and the untagged protein was collected in flow-through with dialysis buffer. These fractions were pooled, concentrated, and buffer exchanged in 1× PBS, pH 8, using a 10 kDa MWCO Amicon centrifugal filter (Millipore Sigma).

Protein concentrations were measured using bicinchoninic acid protein assay (Pierce Protein Assay Kit; Thermo Fisher Scientific). Purity of purified proteins was analyzed by SDS-PAGE gel. Endotoxin levels were measured by the Limulus Amebocyte Lysate Cartridge test (Charles River Laboratories, Wilmington, MA) according to the manufacturer’s instructions.

### Protein-binding and attachment assays

For protein-binding assays, wells of a tissue-culture 96-well plate were coated with 100 μL of 20 μg · mL^−1^ rat tail type I collagen in sterile PBS (Life Technologies, NY), Dpr (40), Cnm, SpaP, PstS, GbpC, GtfD, WapA, or GtfB (Table 2) at 4°C for 18 h. For Cnm preincubation assays, wells were coated with collagen overnight as above, rinsed twice with PBS, and then coated with 2 or 10 μg · mL^−1^ Cnm for 1 h at 37°C in 5% CO_2_. After coating, all wells were gently washed twice with PBS and blocked with 200 μL of PBS containing 0.5% gelatin (wt/vol) for 1 h at 37°C. Starter cultures were centrifuged and resuspended in PBS to OD_600_ 0.5 (*C. albicans* [2 × 10^6^ CFUs]*, S. mutans* [1 × 10^8^ CFUs]*,* or a mixture of *S. mutans* [1 × 10^8^ CFUs] *+ C. albicans* [2 × 10^6^ CFUs]). Cultures were added to quadruplicate wells of each experimental condition and incubated for 1 h at 37°C in 5% CO_2_. Following one wash with PBS, wells were stained with 200 μL of 0.1% crystal violet (wt/vol), washed three times with PBS, and then eluted with 200 μL of a 33% solution of acetic acid (vol/vol). Absorbance was read at 575 nm on a Synergy H1 hybrid multimode reader (BioTek). As a background control, absorbance was measured in wells containing collagen only (no microbes) and subtracted from test values.

For attachment assay with pre-coaggregation in filter-sterilized saliva following PBS rinse as above, cultures were centrifuged and resuspended in saliva to an OD_600_ of 0.5 and then mixed in a 1:2 ratio (*S. mutans: C. albicans*). For single-species controls, the same total mixing volume was maintained by adding the corresponding volume of filter-sterilized saliva instead of the partner culture. Cultures were incubated at 37°C and 5% CO_2_ for 1 h prior to being transferred to quadruplicate wells and continuing as above for all attachment assays.

### Coaggregation assay

To determine coaggregation, *C. albicans* and *S. mutans* starter cultures grown as above were harvested by centrifugation at 4,000 × *g* for 10 min, washed twice with PBS, and once using coaggregation buffer (0.1 mM CaCl_2_, 0.1 mM MgCl_2_, 150 mM NaCl, 3.1 mM NaN_3_ dissolved in 1 mM Tris buffer and adjusted to pH 7.0) (41). Cells were then resuspended and standardized in coaggregation buffer to an optical density of 1.0 at OD_600 nm_. For coaggregation in the presence of collagen, 40 μg/mL rat tail type I collagen in sterile PBS (Life Technologies, NY) was added. Each suspension was mixed thoroughly using a vortex mixer for 30 s, and the OD_600 nm_ at time (*t*) = 0 h was measured. The inoculum was incubated at room temperature for 1 h to allow coaggregation, and the OD_600 nm_ was recorded. Sterile coaggregation buffer was used as a blank. Percentage of coaggregation was calculated using the following equation: ([OD600_*t* = 1 h_ − OD600_*t* = 0 h_]/OD600_*t* = 1 h_)*100.

### Fluorescent strain generation

To generate fluorescent strains that could be visualized by confocal microscopy, we obtained the superfolder GFP integrative plasmid pPMZ-P_veg_-sfgfp (hereafter pPMZ-gfp), which integrates at the mtlA-phnA locus and has been shown not to affect surrounding gene expression (42). After receiving the *Escherichia coli* NEB10β strain containing this plasmid, pPMZ-gfp was propagated and isolated with the R Plasmid Miniprep-Classic kit (Zymo Research, Irvine, CA). The plasmid sequence was confirmed by whole-plasmid sequencing using the Illumina platform (Plasmidsaurus, South San Francisco, CA). Then, pPMZ-gfp was used to transform *S. mutans* UA159 and UA159 + *cnm* using the *comX*-Stimulating Peptide (XIP) (43). For *S. mutans* OMZ175 and OMZ175Δ*cnm* transformation with pPMZ-gfp, competence-stimulating peptide (CSP) was used instead of XIP (44). Briefly, for strain OMZ175, overnight cultures were diluted 1:20 into fresh BHI media for 2 h at 37°C in 5% CO_2_ until the OD_600_ reached 0.1. Then, 1 μM CSP (SGSLSTFFRLFNRSFTQALGK) and 500 ng pPMZ-gfp were mixed with 500 μL of the culture. For the UA159 strain, overnight cultures were diluted 1:15 into fresh chemically defined medium (CDM) (45) + 1% glucose and also grown to an OD_600_ of 0.1. Then, 0.8 μM XIP (GLDWWSL) and 500 ng pPMZ-gfp were mixed with 500 μL of the culture. After this, all strains were treated in the same manner—the mixtures were incubated for 5 h in 5% CO_2_ at 37°C before being plated onto BHI agar supplemented with 1 mg mL^−1^ kanamycin and 10 μg mL^−1^ erythromycin. These plates were incubated for 48 h in 5% CO_2_ at 37°C. Colonies were patched onto fresh BHI plates with kanamycin and erythromycin and screened using Colony PCR and Sanger sequencing (Genewiz, South Plainfield, NJ) according to recommended primers Fwd.Seq.mtlA (CAGTCTTAGTCAGGCTTTG) and Rev.Seq.phnA (GATTCCATTCATAAGCACAT), which amplify the genomic region spanning the mtlA–phnA locus targeted for integration. Successful chromosomal integration of pPMZ-gfp produces a 3.7-kb amplicon corresponding to the inserted cassette flanked by the native homology regions, whereas non-integrated strains yield the 5.7-kb wild-type amplicon (44).

### Confocal microscopy for early attachment

To visualize the role of Cnm in early attachment to collagen-coated wells, for all experiments, UA159 + gfp, UA159 + cnm + gfp, OMZ175 + gfp, OMZ175Δcnm + gfp, and SC5314 were grown separately in BHI broth. After 18-h incubation at 37°C in 5% CO_2_, these cultures were centrifuged, and pellets resuspended in sterile PBS to OD_600_ = 0.5 (*C. albicans—*2 × 10^6^ CFUs*, S. mutans*—1 × 10^8^ CFUs). Single- and dual-species cultures were given a 3-h attachment period in PBS on collagen-coated (18 h, 4°C, 20 μg · mL^−1^ rat tail type I collagen in sterile PBS-Life Technologies, NY) or non-coated ibidi µ-slide eight chamber coverslips (Cat. No. 80826). Early biofilms were then rinsed twice with 0.89% saline solution. Prior to imaging, these biofilms were stained for 10 min in the dark using Calcofluor White (83 μM) M2R blue-fluorescence fungal surface labeling reagent (Molecular Probes Inc., Eugene, OR, USA). The fluorophores were sequentially excited (Calcofluor White with 405 nm, Gfp with 488 nm) and detected (Calcofluor White with 426–450 nm, Gfp with 468–552 nm). The confocal images of biofilms were acquired using a Nikon Ti2 confocal microscope and a Nikon C2plus camera equipped with a Plan Apo λ 60× oil objective with sequential illumination, a z-step of 0.5 µm, and a 1-s scanning speed. Images shown are representative of the average of results obtained from six biological replicates (with three images per replicate). For quantification, a maximum intensity projection of the bottom 10 μm of each biofilm was created, and the individual component biovolumes were quantified using the NIS Elements 5.0 Imaging Software.

### Biofilm formation on collagen-coated surfaces

For assessment of biofilm formation, half of the wells of a tissue-culture 96-well plate were coated with 100 μL of 20 μg · mL^−1^ rat tail type I collagen in sterile PBS (Life Technologies, NY) at 4°C for 18 h. The other half of the wells were left uncoated. Following coating, all wells were gently washed twice with PBS. Starter cultures of *C. albicans, S. mutans,* or *S. mutans + C. albicans* were grown and normalized as above. Cultures were then inoculated in (*C. albicans*—5 × 10^4^ CFUs*, S. mutans*—1 × 10^6^ CFUs) biofilm medium (BM [46]) supplemented with either 20 mM glucose (BMG) or 18 mM glucose plus 2 mM sucrose (BMGS). BMG and BMGs cultures were added to six replicate wells of each experimental condition and incubated for 20 h at 37°C in 5% CO_2_. One well for each condition was rinsed 3× with PBS, scraped, and disrupted in 200 μL of PBS and serially diluted for plating on selective media (MSB for *S. mutans* and SAB for *C. albicans*). Remaining wells were washed once with PBS, stained with 200 μL of 0.1% crystal violet (wt/vol), eluted with 33% acetic acid, and OD_575_ was measured. As a background control, OD_575_ was measured in uncoated or collagen-coated only (no microbes) wells and subtracted from test values.

## RESULTS

### *C. albicans* binds to *S. mutans* surface proteins, including Cnm

**Fig 1:**
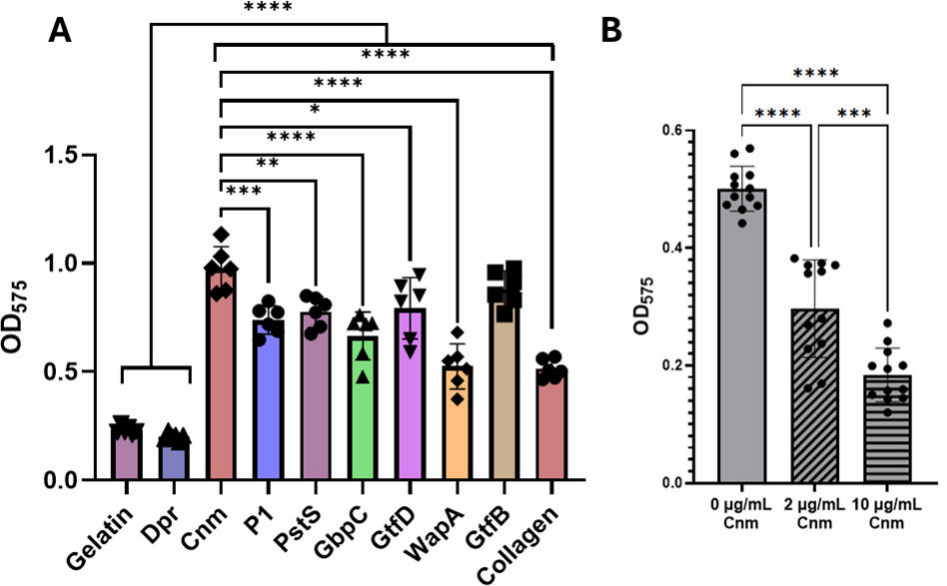
*C. albicans* binds to *S. mutans* surface proteins, including Cnm. (**A**) Protein binding of *C. albicans* to *S. mutans* surface proteins (Cnm, P1, PstS, GbpC, GtfD, WapA, GtfB), a positive control (collagen), and negative controls (gelatin and the intracellular protein Dpr) was assessed using crystal violet staining after a 1-h incubation. (**B**) Collagen-coated wells were preincubated with increasing concentrations of Cnm protein to evaluate the inhibition of *C. albicans* collagen binding. Attachment was quantified by crystal violet staining after a 1-h preincubation and 1-h binding period. Statistical differences were determined by one-way ANOVA with Tukey’s multiple comparisons test. *P < 0.05, **P < 0.01, ***P < 0.001, ****P < 0.0001.

To assess whether *C. albicans* can bind to *S. mutans* selected surface proteins, we performed a protein-binding assay using purified recombinant *S. mutans* surface proteins. Collagen, gelatin, and the intracellular *S. mutans* Dpr were included as controls. Among the surface adhesins tested, GtfB and SpaP were previously reported to interact with *C. albicans* (10, 47). When compared to the negative controls (Dpr and gelatin), we observed that *C. albicans* bound significantly better to collagen and all tested *S. mutans* surface proteins: Cnm, SpaP, PstS, GbpC, GtfD, GtfB, and WapA (*P* < 0.0001) (Fig. 1A). *C. albicans* had more robust binding to Cnm than to collagen, P1, PstS, GbpC, GtfD, or WapA (*P* < 0.05). Similar high levels of binding to Cnm and GtfB by *C. albicans* were noted. Taken together, the data reveal a complex network of interactions by which *C. albicans* recognizes multiple *S. mutans* adhesins. Given that GtfB-mediated binding has been shown to be an important, but sucrose-dependent mechanism of *S. mutans-C. albicans* interaction, we concentrated our subsequent analyses on Cnm, the only other ligand that produced comparably strong binding in our assay, to determine how it mediates collagen- and yeast-directed attachment.

To further define the relationship between Cnm, collagen, and *C. albicans*, we preincubated collagen-coated wells with purified Cnm. Interestingly, Cnm reduced *C. albicans* binding to collagen, indicating that when Cnm is already engaged with collagen, its *C. albicans*–binding interface becomes inaccessible. This suggests that one Cnm protein unit can bind either collagen or *C. albicans*, but not both simultaneously, implying that the two ligands compete for the same or adjacent binding region on Cnm (Fig. 1B). Importantly, Cnm has been shown to be abundant on the surface of expressing *S. mutans* cells; hence, the restriction of a single Cnm protein to bind either Cnm or collagen at one time should not inhibit *S. mutans* cells expressing multiple Cnm units from binding to both collagen and *C. albicans* (Fig. 7).

### Cnm promotes coaggregation between *S. mutans* and *C. albicans*

**Fig 2:**
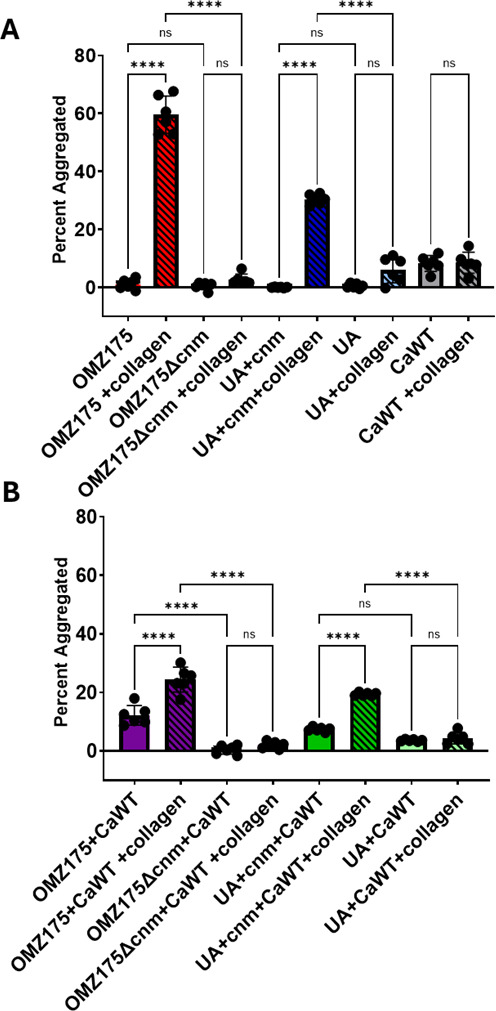
Cnm enhances co-aggregation of *S. mutans* with *C. albicans*. Percent auto-aggregation (**A**) and co-aggregation (**B**) were measured after 1 h of incubation in co-aggregation buffer, either in the presence or absence of 40 µg/mL collagen. Aggregation was calculated using the formula: ([OD600_t = 1 h_ − OD600_t = 0 h_]/OD600_t = 1 h_) × 100. Statistical differences were determined by one-way ANOVA with Tukey’s multiple comparisons test comparing strains with and without *cnm*. *P < 0.05, **P < 0.01, ***P < 0.001, ****P < 0.0001.

*S. mutans* and *C. albicans* are known to coaggregate in saliva, promoting joint adherence and early biofilm development. To test whether Cnm contributes to this interaction, we compared coaggregation among Cnm-positive and Cnm-negative strains: UA159 (*cnm*^−^), OMZ175 (*cnm*^+^), UA159 + cnm (*cnm* knock-in), and OMZ175Δcnm (*cnm* knockout) (Fig. 2). In the absence of collagen, there was no difference in auto-aggregation of *S. mutans* based on the presence of Cnm (Fig. 2A). Additionally, in the absence of collagen, OMZ175 exhibited significantly greater coaggregation with *C. albicans* than its Cnm-deficient derivatives (Fig. 2B). In the presence of collagen, Cnm^+^ strains showed significantly increased auto-aggregation and coaggregation with *C. albicans* relative to their Cnm-deficient counterparts (*P* > 0.05) (Fig. 2A and B). Our findings indicate that *S. mutans* Cnm aids coaggregation with *C. albicans* independently of collagen. However, it appears Cnm-mediated coaggregation is further enhanced in the presence of collagen (*P* < 0.001).

### Cnm enhances attachment of *S. mutans* and dual-species cultures to collagen-coated and uncoated surfaces

**Fig 3:**
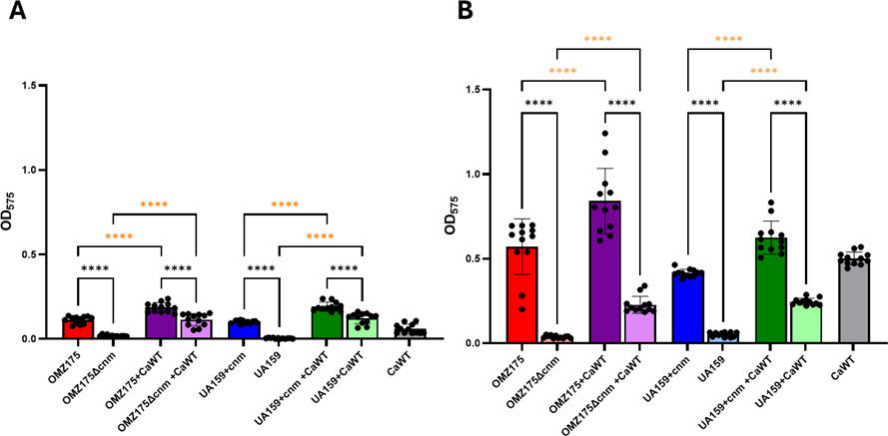
Cnm enhances attachment of single- and dual-species cultures to collagen-coated and uncoated surfaces. Crystal violet staining was used to quantify attachment of single-species (*S. mutans* or *C. albicans*) and dual-species cultures to uncoated (**A**) or collagen-coated (**B**) surfaces following a 1-h incubation. Statistical differences were determined by one-way ANOVA with Tukey’s multiple comparisons test, comparing strains with and without *cnm* (black), and the presence or absence of *C. albicans* (orange). *P < 0.05, **P < 0.01, ***P < 0.001, ****P < 0.0001.

Given the Cnm role in the coaggregation, we next assessed whether Cnm also promotes initial surface attachment. Single-species (*S. mutans*) and dual-species (*S. mutans–C. albicans*) cultures were allowed to adhere to collagen-coated or uncoated wells for 1 h. As a single species, Cnm^+^ strains (OMZ175 and UA159+cnm) showed significantly greater attachment to both uncoated (Fig. 3A) and collagen-coated surfaces (Fig. 3B) compared to Cnm-deficient strains (*P* < 0.0001). Similarly, Cnm significantly promoted attachment of co-cultures to uncoated and collagen-coated surfaces (Fig. 3B). Attachment was also improved for all co-culture conditions compared to the *S. mutans*-only condition (*P* < 0.0001). Interestingly, Cnm-enhanced attachment is further increased in the presence of collagen (*P* < 0.001)

**Fig 4:**
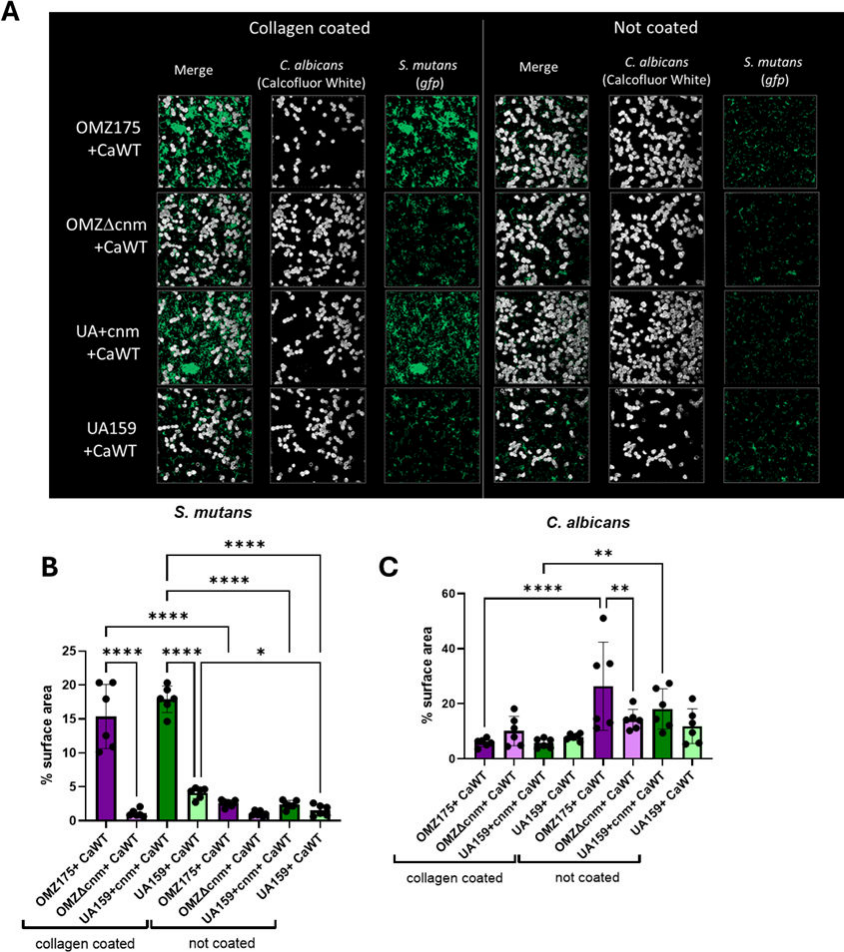
Cnm promotes early attachment of *S. mutans–C. albicans* biofilms. (**A**) Representative confocal microscopy images of dual-species biofilms on uncoated and collagen-coated surfaces after a 3-h attachment period. Green = GFP-expressing *S. mutans*; white = *C. albicans* stained with Calcofluor White. Each panel shows a top-down 3D reconstruction of the biofilm. (**B and C**) Biovolume quantification of each species was performed using maximum intensity projections of the bottom 10 µm of the biofilm to calculate the percent surface area covered. Statistical analysis was performed using one-way ANOVA with Tukey’s multiple comparisons test. *P < 0.05, **P < 0.01, ***P < 0.001, ****P < 0.0001.

Confocal microscopy showed that Cnm enhanced *S. mutans* single-species attachment in the presence and absence of collagen and for UA159 + cnm on uncoated surfaces (Fig. S1). Fig. 4A shows that Cnm promotes early attachment of *S. mutans–C. albicans* biofilms. Importantly, in dual species biofilms, Cnm enhanced *S. mutans* attachment to collagen-coated surfaces when compared to Cnm-deficient strains (Fig. 4B). For *C. albicans*, Cnm promoted recruitment into biofilms only on uncoated surfaces (Fig. 4C).

### Pre-coaggregation in saliva enhances dual-species Cnm-dependent attachment

**Fig 5:**
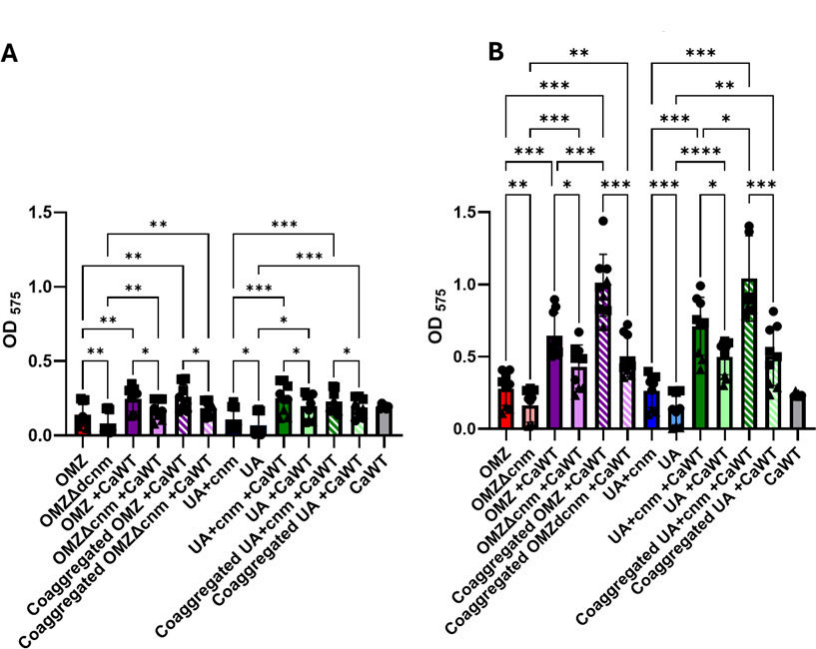
Cnm and prior coaggregation enhance attachment of single- and dual-species cultures to collagen-coated and uncoated surfaces. *S. mutans* and *C. albicans* were individually or coaggregated in saliva for 1 h followed by an additional 1-h attachment period. Crystal violet staining was used to quantify attachment of single-species (*S. mutans* or *C. albicans*), dual-species (aggregated individually), and dual-species (coaggregated) cultures to uncoated (**A**) or collagen-coated (**B**) surfaces. Statistical differences were determined by one-way ANOVA with Tukey’s multiple comparisons test, comparing strains with and without *cnm*, the presence or absence of *C. albicans,* and coaggregation status. *P < 0.05, **P < 0.01, ***P < 0.001, ****P < 0.0001.

To test whether pre-formation of *S. mutans–C. albicans* coaggregates alters subsequent ability to attach to surfaces, we pre-incubated *S. mutans* and *C. albicans* in filter-sterilized pooled human saliva for 1 h to allow co-aggregation or individual aggregation, then transferred the suspensions to collagen-coated wells and quantified attached biomass by crystal violet staining after 1 h. As a single species, Cnm^+^ strains (OMZ175 and UA159 + cnm) showed significantly greater attachment to both uncoated (Fig. 5A) and collagen-coated surfaces (Fig. 5B) compared to Cnm-deficient strains (*P* < 0.05). Cnm significantly promoted attachment of individually aggregated co-cultures to uncoated and collagen-coated surfaces (*P* < 0.05) (Fig. 5A and B). Cnm also increased attachment of co-aggregated *S. mutans* and *C. albicans* cultures for both uncoated and coated surfaces (Fig. 5). Interestingly, Cnm-enhanced attachment is further increased in the presence of collagen (*P* < 0.001). Both dual-species conditions (pre-coaggregated and individually aggregated) demonstrated enhanced attachment compared to the *S. mutans*-only condition (*P* < 0.05). Furthermore, on collagen surfaces coaggregated co-cultures of Cnm + *S. mutans* and *C. albicans* demonstrated further enhancement of attachment (*P* < 0.05) (Fig. 5B). These coaggregated wells also demonstrated larger visible aggregates following staining (representative images in supplementary Fig. S2). Thus, when S. *mutans and C. albicans* cells were allowed to co-aggregate in saliva before being placed on collagen, coaggregation of *C. albicans* with Cnm-positive *S. mutans* resulted in greater attachment than non-previously coaggregated controls, indicating that the abundance of surface-exposed Cnm molecules allows cells to bind collagen and *C. albicans* at the same time, consistent with the dual-interaction model shown in Fig. 7.

### Cnm enhances 24-h single- and dual-species biofilms on collagen-coated surfaces

**Fig 6:**
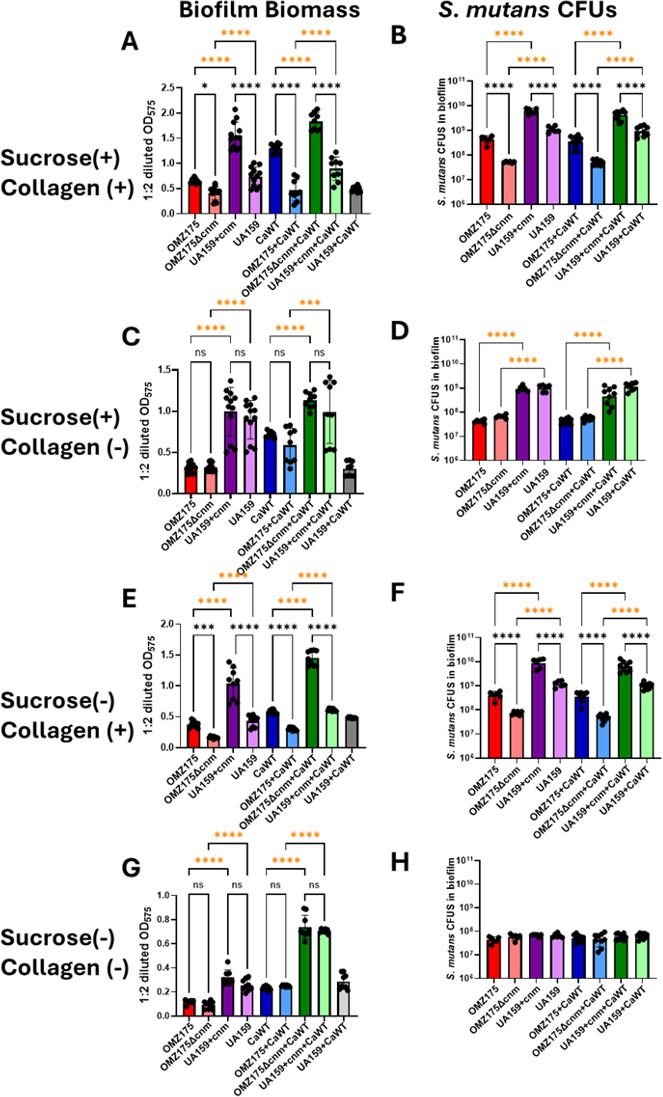
Cnm, sucrose, and collagen promote 24-h biofilm formation by *S. mutans* and *C. albicans*. Biofilms were grown for 24 h under four conditions: collagen-coated or uncoated surfaces, with or without 2 mM sucrose. Biofilm biomass (**A, C, E, and G**) was quantified by crystal violet staining. CFUs were determined via selective plating for *S. mutans* (**B, D, F, and H**) and *C. albicans* (Fig. S3). Statistical differences were evaluated using one-way ANOVA with Tukey’s multiple comparisons test, comparing strains with and without *cnm* (black), and the presence or absence of *C. albicans* (orange). *P < 0.05, **P < 0.01, ***P < 0.001, ****P < 0.0001.

Here, we sought to determine the contribution of Cnm in sucrose-rich (cariogenic condition) or free environments to biofilm formation on collagen-coated wells utilizing *S. mutans* (UA159, OMZ175, and their *cnm*-altered derivatives) alone or in dual-species culture with *C. albicans* (Fig. 6). Across all strains and conditions, biomass was significantly increased by the addition of sucrose, collagen coating, and Cnm expression (three-way ANOVA, *P* < 0.0001). The combined presence of collagen and Cnm expression significantly enhanced biomass in both single- and dual-species biofilms (three-way ANOVA *P* < 0.001). When media conditions were analyzed individually, Cnm expression significantly increased biofilm biomass and *S. mutans* CFUs on collagen-coated plates, regardless of sucrose availability (Fig. 6A, B, E and F). In contrast, no significant increase in biomass or CFUs was observed on uncoated surfaces, suggesting that Cnm’s effect on 24-h biofilms is dependent on the presence of collagen (Fig. 6C, D, H and G). Interestingly, while the presence of sucrose in collagen-free plates resulted in increased biomass, there was no corresponding increase in *S. mutans* CFUs, consistent with an increase in glucan production by glucosyltransferases in the presence of sucrose (Fig. 6C and D). Contrastingly, in collagen-containing environments, both in the presence and absence of sucrose, there is a marked increase in both biomass and *S. mutans* CFUs (Fig. 6A, B, E and F), consistent with increased *S. mutans* recruitment to the biofilm via increased adhesion.

In all tested conditions, dual-species biofilms were more robust than single-species biofilms formed (Fig. 5A, C, E, and G). *S. mutans* CFUs were also consistently higher in dual-species cultures, except under no-sucrose, uncoated conditions (Fig. 5B, D, F, and H). *C. albicans* CFUs were increased specifically in the collagen-coated, no-sucrose condition in the presence of OMZ175, OMZ175Δcnm, or UA159 +cnm (Fig. S3).

## DISCUSSION

Dental caries is a microbially mediated biofilm-driven polymicrobial disease characterized by enamel dissolution due to microbial carbohydrate metabolism that releases acid as a byproduct. While *S. mutans* alone is capable of shifting the oral microbial community toward a more cariogenic state, the mere presence of *S. mutans* in saliva is not a reliable predictor of caries risk (48, 49). However, the colonization with *S. mutans* strains harboring bona fide Cbps, such as Cnm or Cbm, has been linked to increased caries risk and early childhood caries (ECC) (21, 22, 50, 51). Importantly, colonization with Cbp^+^ *S. mutans* results in an increased burden of *S. mutans* and *C. albicans* in carious dentin (22). Here, we investigated whether Cnm can mediate *C. albicans* recruitment to the growing biofilms in the presence of collagen under cariogenic conditions and showed evidence that *C. albicans* directly binds to Cnm and other surface-associated proteins of *S. mutans*.

*C. albicans* forms physical associations with several oral bacterial species through specific adhesin interactions that facilitate coaggregation and biofilm development. One of the best-characterized mechanisms involves the fungal adhesin Als3 and Hwps1, which bind directly to SspB, a surface protein of *Streptococcus gordonii*, enabling tight interspecies adherence and mixed biofilm formation (52). The broad-spectrum *C. albicans* adhesin Eap1 also contributes to early-stage coaggregation with *Actinomyces naeslundii* and *Fusobacterium nucleatum*, supporting initial colonization and biofilm layering with bacterial partners (53). Additionally, *C. albicans* cell wall mannoproteins, particularly those modified by Mnt1/Mnt2 mannosyltransferases, present high-affinity binding sites for bacterial lectin-like adhesins, facilitating interactions with *Veillonella* and *Fusobacterium* spp. (53). In our protein-binding assay, we demonstrated that *Candida albicans* efficiently binds to multiple *S. mutans* surface proteins. Among all tested targets, *C. albicans* showed the most robust binding to Cnm and GtfB. The well-characterized interaction between GtfB and *C. albicans* involves GtfB binding to the mannans present in the fungal cell wall (9, 10, 54). The observation that *C. albicans* binds as robustly to Cnm as to GtfB is a strong indication that, when present, Cnm may play an important and unrecognized role in *S. mutans* binding to *C. albicans*. We also validated known interactions of *C. albicans* with collagen (55), P1 (SpaP) (12), and GtfD (9). We found that *C. albicans* can bind to the phosphate-binding protein PstS that is part of a phosphate ABC transporter complex. Deletion of the gene coding for PstS (*pstS*) in *S. mutans* has been shown to reduce binding to the salivary pellicle and reduce EPS production; moreover, PstS plays an important role in tooth colonization *in vivo* (37, 56). Here, we show that PstS also serves as a binding target for *C. albicans,* but it remains unknown whether it alters PstS function. Across the board, we see that *C. albicans* has a remarkable affinity for *S. mutans* adhesins, supporting a model in which multiple *S. mutans* adhesins contribute to *C. albicans* recognition, but their relative importance likely varies with environmental conditions. GtfB–mannan interactions are well established in sucrose-rich settings, whereas Cnm provides a sucrose-independent, collagen-targeted mechanism that may be especially relevant in deeper collagen-exposed niches such as dentin, where sucrose is not as abundant as it is in environmentally exposed enamel surfaces. Thus, Cnm should be viewed as a context-specific adhesin whose contribution becomes most apparent when sucrose is limited or collagen is abundant. Yet, further investigation is needed to uncover which *C. albicans* elements recognize these *S. mutans* proteins. While *C. albicans* Als1 and Hwp1 have been shown to play a role in *S. mutans–C. albicans* adherence in the Cnm^−^ strain UA159, there are no confirmed *S. mutans* binding partners for these proteins (57). Further studies are required to determine whether any of the proteins identified here serve as binding partners for Als1 or Hwp1 and whether there are still additional mechanisms by which *C. albicans* recognizes these *S. mutans* adhesins.

By utilizing a collagen-coated plate that was then coated with Cnm before *C. albicans* introduction, we observed a Cnm abundance-dependent decrease in *C. albicans* binding. Cnm has been well established to bind collagen through its CBD. The reduction in *C. albicans* binding following Cnm incubation suggests that with the CBD oriented to bind to collagen, *C. albicans* binding is not simultaneously possible (33, 34). Importantly, it is known that microorganisms, specifically *C. albicans,* express collagen-like mannoproteins that mimic certain domains of collagen, as well as Als and Hwp proteins, which are known to have roles in other bacterial-*Candida* binding (58, 59). However, whether Cnm recognizes any of these proteins or another *C. albicans* surface-associated ligand is currently unknown.

*In vivo* studies have shown that microbial aggregates formed either by aggregation in saliva or detachment from existing biofilms serve as primary colonizers in biofilm formation. Specifically, *S. mutans–C. albicans* assemblies can adhere together and initiate biofilm formation more rapidly than either species individually (11). In our coaggregation assays, we found that Cnm-expressing *S. mutans* strains (OMZ175 and UA159 + *cnm*) coaggregate more readily with *C. albicans* than their Cnm-negative counterparts. This enhanced coaggregation occurred even in the absence of collagen, suggesting that Cnm mediates direct interaction with *C. albicans* that is independent of collagen. However, this aggregation was further enhanced in a collagen-containing environment, likely due to enhanced auto-aggregation of *S. mutans* in conjunction with the coaggregation occurring with *C. albicans*.

In the oral environment, biofilms must continually reestablish following routine disturbances such as food intake or oral hygiene. If Cnm expression enhances the ability of *S. mutans* and *C. albicans* to initiate early attachment, this advantage could enable these dysbiosis-associated partners to compete more effectively against health-associated commensals during the earliest stages of biofilm redevelopment. We found that Cnm significantly enhances early biofilm attachment in both single-species and dual-species cultures. On collagen-coated surfaces, this enhancement was driven primarily by increased *S. mutans* adherence, consistent with the established role of Cnm as a collagen-binding adhesin. On uncoated surfaces, however, enhanced attachment was largely attributable to increased *C. albicans* association, particularly with strain OMZ175, supporting the idea that fungal cells can engage Cnm directly. Importantly, because our confocal microscopy and attachment assays were performed under conditions that maintain *C. albicans* mostly in the yeast form, the enhanced association observed here reflects mainly *S. mutans–*yeast (not hyphae) interactions. Importantly, imaging of *C. albicans* from carious lesions has shown that acidic ECC environments suppress hyphal formation and favor yeast-dominant populations (11, 30–32). However, future work should investigate how fungal morphological state influences *S. mutans–C. albicans* interactions.

Our saliva-based coaggregation–adhesion assay further supports the idea that Cnm enhances early colonization by strengthening initial physical associations between *S. mutans* and *C. albicans*. Pre-formed dual-species coaggregates generated in saliva adhered more efficiently to both collagen-coated and uncoated surfaces when Cnm was present, indicating that Cnm not only promotes coaggregation but also increases the likelihood that these aggregates successfully transition to surface-attached communities. These findings indicate that Cnm can promote early colonization through interactions with either collagen or *C. albicans*, even though our protein-binding assays demonstrate that an individual Cnm molecule cannot engage both ligands simultaneously. Consequently, in collagen- and sucrose-deficient environments, the basis for the enhanced early attachment is likely more complex. Early dual-species attachment likely arises from multiple, potentially concurrent processes: Cnm^+^ *S. mutans* may initiate surface attachment and subsequently have enhanced recruitment of *C. albicans; C. albicans* may adhere first and facilitate the accumulation of Cnm^+^ *S. mutans*; or both events may occur. Given that *C. albicans* can engage several *S. mutans* adhesins (e.g., SpaP), Cnm is best understood as one component of a broader adhesin network that collectively supports early co-colonization.

Finally, we evaluated 24-h biofilm formation under varying conditions, including the presence or absence of sucrose and collagen. As expected, and in line with the known role of *C. albicans* in upregulating *S. mutans gtf* genes and promoting EPS production, both sucrose and *C. albicans* presence independently enhanced *S. mutans* biomass. Importantly, expression of Cnm further enhanced biomass and *S. mutans* CFUs, but only on collagen-coated surfaces. This increase aligns with the findings from our lab’s clinical study on ECC patients. Even among recurrent caries patients, those patients with Cbp^+^ *S. mutans* strains had an increased *S. mutans* carriage in plaque from dentin, which is composed of collagen. These same patients did not have elevated *S. mutans* levels from enamel plaque samples, mirroring our results from non-collagen surfaces (22). This suggests that Cnm-mediated advantages in initial attachment may translate into more robust long-term biofilm development in collagen-rich environments, such as exposed dentin. On non-coated surfaces, the Cnm-dependent effect is no longer observed, and enhanced biofilm biomass and *S. mutans* CFUs are likely driven by different mechanisms, like glucan production by Gtfs in the presence of sucrose and by the role of SpaP in biofilm development in the absence of sucrose.

**Fig 7:**
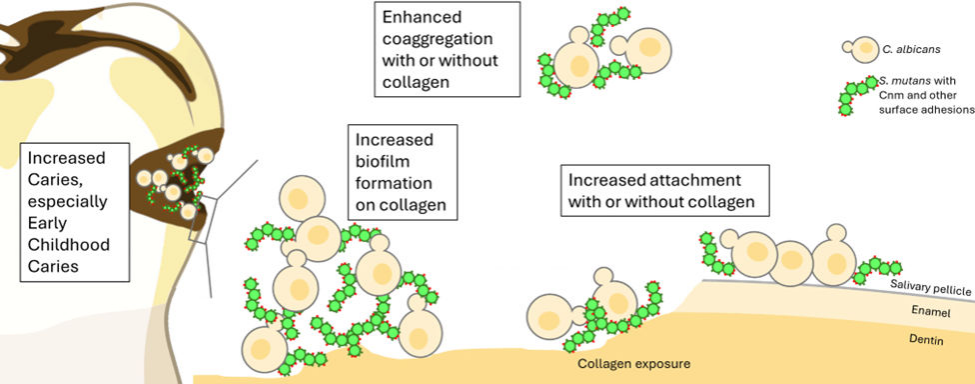
Proposed model of Cnm-mediated enhancement of *S. mutans–C. albicans* biofilm formation. Cnm promotes co-aggregation between *S. mutans* and *C. albicans*, enhances surface attachment, particularly to collagen, and supports increased biomass of dual-species biofilms on collagen surfaces. Together, these effects may contribute to the heightened cariogenic potential observed in clinical studies for those harboring both organisms.

In conclusion, our findings suggest that Cnm plays a pivotal role in promoting interactions between *S. mutans* and *C. albicans*, thereby expanding the current paradigm of synergism between these two microorganisms, especially in the presence of collagen (Fig. 7). Our results highlight the clinical and environmental significance of Cnm^+^ *S. mutans* strains in increasing caries risk and fostering the development of robust and hypervirulent biofilms.

## SUPPLEMENTAL MATERIAL

Supplemental material aem.01046-26-s0001.docx Fig. S1 to S3.

ASM does not own the copyrights to Supplemental Material that may be linked to, or accessed through, an article. The authors have granted ASM a non-exclusive, world-wide license to publish the Supplemental Material files. Please contact the corresponding author directly for reuse.
