# Supplementary material for: The Streptococcus mutans collagen-binding protein Cnm enhances early biofilm formation with Candida albicans
Source: Appl Environ Microbiol. 2026 Jun 30;92(7):e01046-26. doi: 10.1128/aem.01046-26 (PMC13390344; doi:10.1128/aem.01046-26)
Supplement: Supplemental material — Fig. S1 to S3. [file aem.01046-26-s0001.docx]

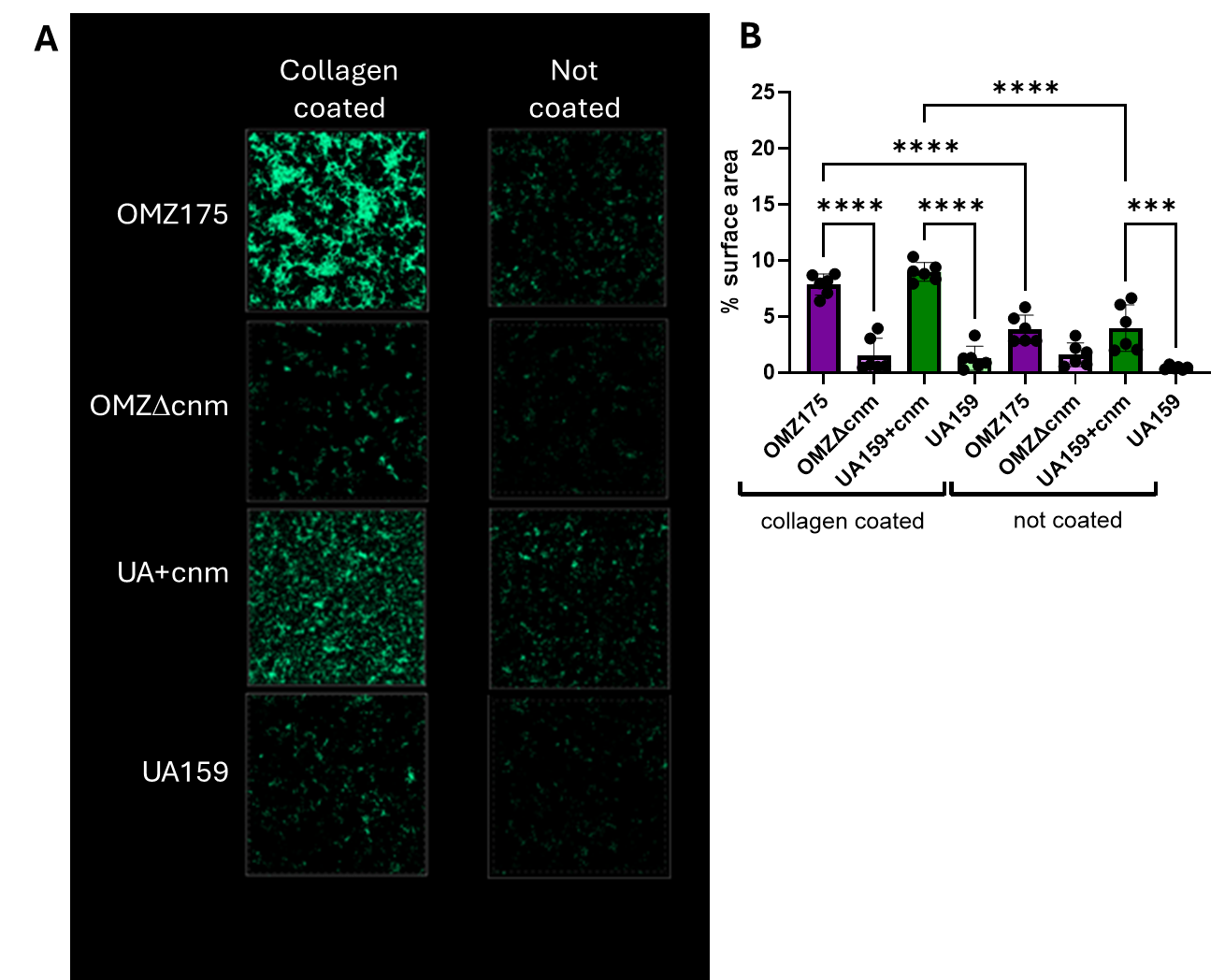


Figure S1. Cnm promotes early attachment of *S. mutans* biofilms to collagen surfaces. (A) Representative confocal microscopy images of single-species biofilms on uncoated and collagen-coated surfaces after a 3-hour attachment period. Green = GFP-expressing *S. mutans*; Each panel shows a top-down 3D reconstruction of the biofilm. (B) Biovolume quantification was performed using maximum intensity projections of the bottom 10 µm of the biofilm to calculate the percent surface area covered. Statistical analysis was performed using one-way ANOVA with Tukey’s multiple comparisons test. *p<0.05, **p<0.01, ***p<0.001, ****p<0.0001.


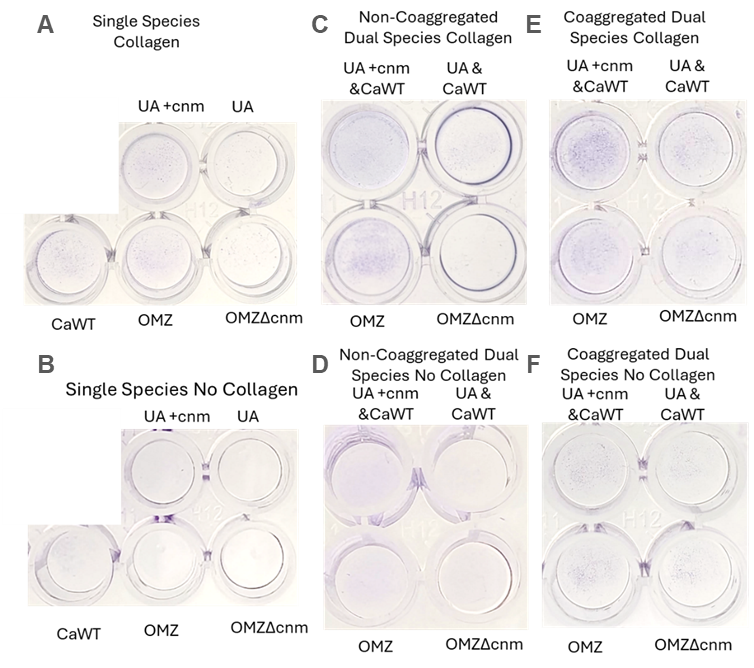


Figure S2. Cnm and prior coaggregation enhance attachment of single and dual-species cultures to collagen-coated and uncoated surfaces. *S. mutans* and *C. albicans* were individually or coaggregated in saliva for 1hr followed by an additional 1hr attachment period. Crystal violet staining was used to assess attachment of single-species (*S. mutans* or *C. albicans*)(A&B), dual-species (aggregated individually)(C&D), and dual-species (coaggregated)(E&F) cultures to uncoated (A,C,E) or collagen-coated (B,D,F) surfaces. Following rinsing and staining, photos of representative wells were taken to visualize the attachment of aggregated microorganisms.


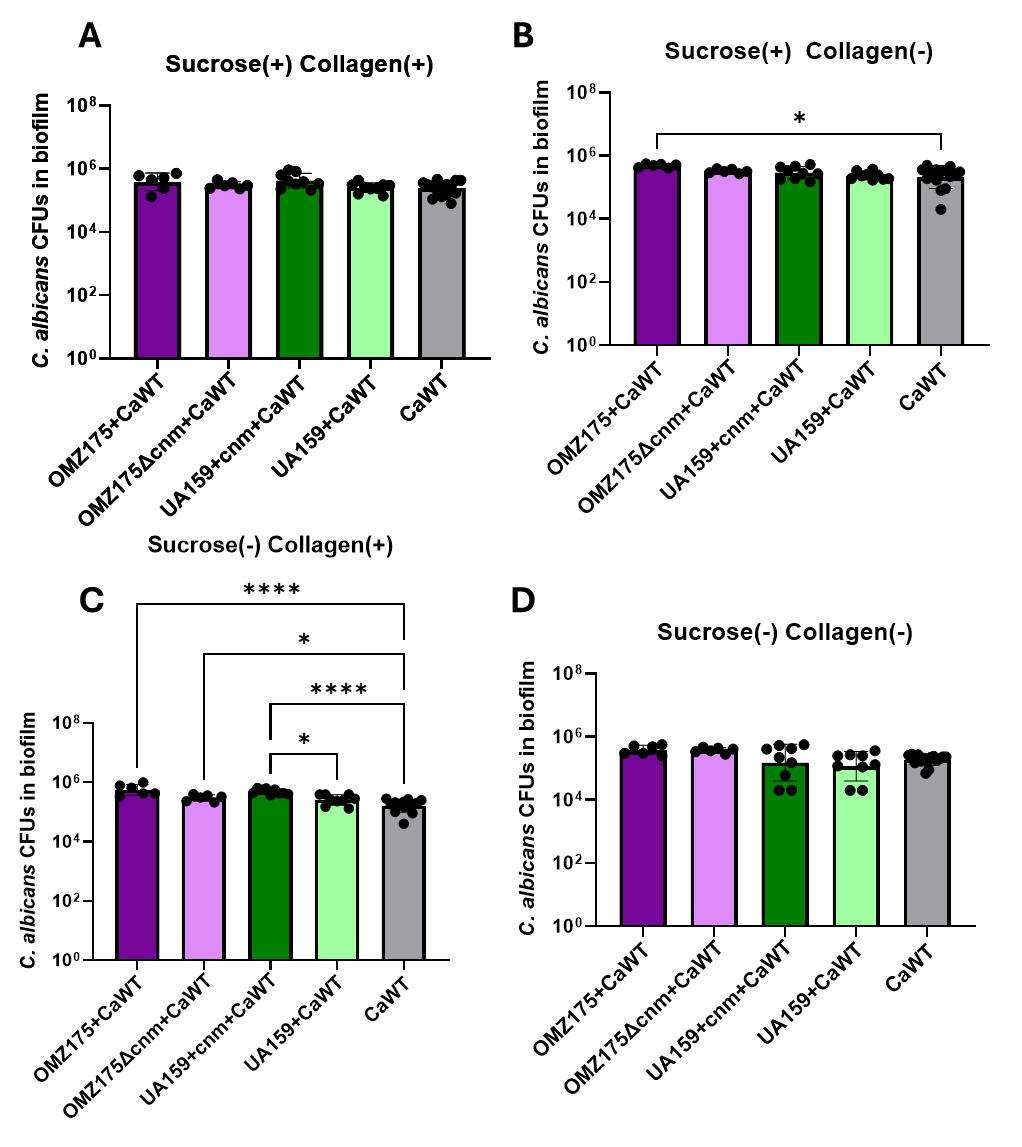


Figure S3. Cnm, sucrose, and collagen promote 24-hour biofilm formation by *S. mutans* and *C. albicans*. Biofilms were grown for 24 hours under four conditions: collagen-coated or uncoated surfaces, with or without 2 mM sucrose. Biofilm biomass (Figure 5) was quantified by crystal violet staining. CFUs were determined via selective plating for *S. mutans* (Figure 5) and *C. albicans* (Figure A,B,C,D). Statistical differences were evaluated using one-way ANOVA with Tukey’s multiple comparisons test. *p<0.05, **p<0.01, ***p<0.001, ****p<0.0001.
